# Episodic memory dysfunction and hypersynchrony in brain functional networks in cognitively intact subjects and MCI: a study of 379 individuals

**DOI:** 10.1007/s11357-022-00656-7

**Published:** 2022-09-15

**Authors:** Brenda Chino, Pablo Cuesta, Javier Pacios, Jaisalmer de Frutos-Lucas, Lucía Torres-Simón, Sandra Doval, Alberto Marcos, Ricardo Bruña, Fernando Maestú

**Affiliations:** 1 Institute of Neuroscience, Autonomous University of Barcelona, Barcelona, Spain; 2 Center for Cognitive and Computational Neuroscience, Complutense University of Madrid, Madrid, Spain; 3 Department of Radiology, Rehabilitation, and Physiotherapy, Complutense University of Madrid, Madrid, Spain; 4 Instituto de Investigación Sanitaria San Carlos (IdISSC), Madrid, Spain; 5 Department of Experimental Psychology, Complutense University of Madrid, Madrid, Spain; 6 Centre for Precision Health, Edith Cowan University, Joondalup, WA 6027 Australia; 7 Neurology Department, Hospital Clinico San Carlos, Madrid, Spain; 8 Centro de Investigación Nebrija en Cognición (CINC), Universidad de Nebrija, Madrid, Spain

**Keywords:** Delayed recall, MCI, Cognitively normal, Functional connectivity, MEG

## Abstract

Delayed recall (DR) impairment is one of the most significant predictive factors in defining the progression to Alzheimer’s disease (AD). Changes in brain functional connectivity (FC) could accompany this decline in the DR performance even in a resting state condition from the preclinical stages to the diagnosis of AD itself, so the characterization of the relationship between the two phenomena has attracted increasing interest. Another aspect to contemplate is the potential moderator role of the APOE genotype in this association, considering the evidence about their implication for the disease. 379 subjects (118 mild cognitive impairment (MCI) and 261 cognitively intact (CI) individuals) underwent an extensive evaluation, including MEG recording. Applying cluster-based permutation test, we identified a cluster of differences in FC and studied which connections drove such an effect in DR. The moderation effect of *APOE *genotype between FC results and delayed recall was evaluated too. Higher FC in beta band in the right occipital region is associated with lower DR scores in both groups. A significant anteroposterior link emerged in the seed-based analysis with higher values in MCI. Moreover, *APOE* genotype appeared as a moderator between beta FC and DR performance only in the CI group. An increased beta FC in the anteroposterior brain region appears to be associated with lower memory performance in MCI. This finding could help discriminate the pattern of the progression of healthy aging to MCI and the relation between resting state and memory performance.

## Introduction

Alzheimer’s disease (AD) is a progressive neurodegenerative disorder that can develop unnoticed for several years prior to the manifestation of clinical symptomatology. Along with executive functioning, episodic memory impairments are the hallmark of the cognitive dysfunction associated with the disease [1, 2], which accompany histopathological and morphological changes. In particular, delayed recall (DR) of meaningful information is notably affected in AD, with recall of stories showing the sharpest deterioration in longitudinal studies [3]. Moreover, episodic memory declines are well documented in people not yet diagnosed with AD [4] (see also Bäckman et al. [5] for meta-analytic evidence), suggesting that individual performance on tests evaluating this cognitive domain may help to identify people either at risk of developing AD or even in prodromal stages as mild cognitive impairment (MCI) [3]. Moreover, the genotype for the apolipoprotein E (*APOE*) is known to be the major genetic risk factor for AD, and carriers of ε4 allele show greater impairments on episodic memory performance in the form of poorer DR (e.g., Wolk et al. [6]).

On the neurophysiological level, the last decades have seen the emergence of functional connectivity (FC) and its identification as a potential neuromarker for the diagnosis of AD [7, 8]. Disrupted synchronization is considered as a sign of synaptic dysfunction in AD, consequence of abnormal neural excitation/inhibition balance [9]. Indeed, people with MCI tend to show hypersynchronization of anterior networks, in combination with hypo-synchronization of posterior areas [10, 11]. Given that episodic memory impairments could be accompanied by changes in brain FC from the preclinical stages of the disease to the diagnosis of AD itself, the characterization of the functional relationship between the two phenomena has attracted increasing interest. Rather than focusing on task-related FC measures, which can be affected by variability in experimental designs and task difficulty [12], neuroimaging research in the last years has focused on the study of resting-state (RS) functional connectivity patterns and how they relate to individual differences in cognitive performance (e.g., del Río et al. [13]). Indeed, increased FC, measured during functional magnetic resonance imaging (fMRI), has been associated with reduced episodic memory in patients with MCI and AD [14]. However, fMRI-based FC estimates are an indirect measure of brain activity. Furthermore, they provide limited information on the frequency of neural oscillations, particularly those in the fast ranges, considered one of the core neural mechanisms for cognition, and more relevant to this study, for episodic memory [15, 16]. Therefore, neurophysiological measures such as magnetoencephalography (MEG) or electroencephalography (EEG), having a high temporal resolution, are able to capture the brain dynamics in the frequency domain providing an enriched information for network analysis.

Considering all the evidence provided above, in this study we explore the potential association between delayed recall performance and FC of resting-state brain activity in healthy volunteers and MCI patients. As a second step, the role of *APOE* genotype as a potential moderator of the relation between FC and delayed recall was evaluated, under the hypothesis that this genotype can increase brain vulnerability to damage. We expect that subjects with MCI will show a differential functional connectivity profile than cognitively intact (CI) participants related to memory performance, indicating preliminary signs of conversion in the AD continuum.

## Methods

### Participants

The sample consisted of 379 individuals divided into 118 MCI (aged from 58 to 87) and 261 CI participants (aged from 41 to 82). The participants were recruited from the Hospital Universitario San Carlos [17] and from “Centro para Mayores del Distrito de Chamartín”, both located in Madrid (Spain). General inclusion criteria were as follows: a modified Hachinski score ≤ 4, a Geriatric Depression Scale (short form) score ≤ 5, and T1, T2, and diffusion-weighted MRIs within 54 weeks before the MEG recordings (on average, the time period between the MEG and MRI recordings was 3 months) without an indication of infection, infarction, or focal lesions (rated by two independent experienced radiologists [18]). In addition, the criteria for the MCI diagnosis were established according to the NIA-AA clinical criteria [19]. For more information about the diagnostic criteria for MCI, see López et al. [20]. For CI participants, we exclude subjects with evidence of significant hippocampal atrophy in a T1-weighted MRI scan within 2 months before MEG acquisition, as hippocampal atrophy is considered a brain marker associated with neurodegeneration [21]. No one of the participants exhibited a history of psychiatric or neurological disorders other than MCI. Furthermore, we advised subjects to avoid medications that could affect MEG activity, such as benzodiazepines, for 48 h before recordings (A detailed list of the sample characteristics can be found in Table 1).

**Table 1:** Descriptive measures of the final sample

Variable	Whole sample	CI	MCI	*p* values
Sex (M/F)	135/244	91/170	44/74	
Age	68.00 ± 8.59	65.96 ± 8.48	74.28 ± 5.26	< 0.001
Education	13.24 ± 5.54	14.30 ± 5.49	9.99 ± 4.33	< 0.001
Depression	2.23 ± 2.74	1.63 ± 2.33	4.05 ± 3.09	< 0.001
Delayed recall	40.46 ± 23.21	49.98 ± 17.18	11.14 ± 12.20	< 0.001
eTIV	1,384,457.31 ± 8190.08	1,395,275.80 ± 154,063.19	1,359,214.17 ± 148,921.36	0.224
Total GM	558,888.08 ± 545,633.32	567,118.08 ± 53,093.03	533,530.78 ± 51,447.00	< 0.001
Total WM	424,346.04 ± 64,360.58	432,579.60 ± 64,658.18	398,977.79 ± 56,705.69	< 0.001
Hippocampus	6829.66 ± 952.96	7132.30 ± 846.21	6123.50 ± 805.16	< 0.001
Left cingulum	0.41 ± 0.04	0.41 ± 0.03	0.39 ± 0.04	< 0.001
Right cingulum	0.41 ± 0.40	0.41 ± 0.03	0.39 ± 0.04	< 0.001
Forceps major	0.55 ± 0.03	0.55 ± 0.03	0.53 ± 0.04	< 0.001
Forceps minor	0.54 ± 0.04	0.54 ± 0.04	0.52 ± 0.03	< 0.001

We present values as mean ± standard deviation (SD) for the characteristics of the participants as well as variables used for correlation analyses. These include sex (male/female), age (in years), education (expressed in years of education), depression (measured by global depression scale), delayed recall (Wechsler Scale-Logical Memory II Index) and structural measures such as *eTIV* estimated total intracranial volume, *GM* total gray matter volume, *WM* cerebral white matter volume, hippocampus volume, left and right cingulum along the hippocampal cortex fractional anisotropy, forceps major fractional anisotropy, and forceps minor fractional anisotropy. Results are displayed for the whole sample and for each subsample of interest (CI and MCI)

### Standard protocol approvals, registrations, and patient consents

All participants were native Spanish speakers and provided written informed consent. The Institutional Review Board Ethics Committee at Hospital Universitario San Carlos approved the study protocol, and the procedure was performed following the Helsinki Declaration and National and European Union regulations.

### Neuropsychological assessment

All participants were screened using standardized diagnostic instruments and received a thorough neuropsychological assessment as formerly detailed in López et al. [20]. The screening consisted of standardized tests that included the Spanish version of the Mini-Mental State Examination (MMSE; [22]), the Geriatric Depression Scale-Short Form (GDS-SF; [23]), and the Logical Memory (I and II) subtest (Wechsler Memory Scale III, WMS-III; [24]).

Due to its effectiveness as a measure of verbal episodic memory, logical memory (LM) is one of the most frequently administered subtests in the Wechsler Memory Scale-III (LM-WMS-III) [24]. In the LM test, the participants presented a text, and the memory ability is divided into immediate recall, delayed recall, and recognition. Our study only included the analysis of the delayed recall score, which consisted of free recall of the passages after a 20 to 30 min delay after the presentation. The narrative nature of the task is sensitive to discriminate between normal aging, MCI [25], and early dementia, due to its tight relationship with other high-level cognitive functions such as episodic memory, conceptual organization, and schema formation [26].

### MRI acquisition and volumetric analyses

We used a General Electric 1.5 T system with a high-resolution antenna and a homogenization PURE filter (Fast Spoiled Gradient Echo sequence, TR/TE/TI = 11.2/4.2/450 ms; flip angle 12°; 1-mm slice thickness, 256 × 256 matrix, and FOV 25 cm) to obtain T1-weighted images of our participants. The resulting images were processed using the Freesurfer software (version 5.1.0) and its specialized tool for automated cortical parcellation and subcortical segmentation [27]. The measures that were included in further analyses were total gray matter, total cerebral white matter, and hippocampus (in mm^3^). The volumes of bilateral structures were collapsed in order to obtain a single measure for each region.

### Diffusion tensor imaging

The same scanner was also used to collect diffusion-weighted images (DWI) (single-shot echo planar sequence, TE/TR 96.1/12,000 ms; NEX 3 for increasing the SNR; 2.4-mm slice thickness, 128 × 128 matrix, and 30.7 cm FOV). We acquired 1 image with no diffusion sensitization (i.e., b0 images) and 25 DWI directions (*b* = 900 s/mm^2^).

DWI images were processed using probabilistic fiber tractography which was run on the automated tool AutoPtx (https://fsl.fmrib.ox.ac.uk/fsl/fslwiki/AutoPtx) as in Verdejo-Román et al. [28]. Due to its relation to memory performance, we studied the relation between FC, DR, and fractional anisotropy (FA) at the uncinate, forceps major, and forceps minor.

### Magnetoencephalography

MEG data was recorded using a 306-channel whole-head MEG system (Vectorview, Elekta AG, Finland), placed in a magnetically shielded room located at the Center for Biomedical Technology in Madrid, following the protocol described in de Frutos-Lucas et al. [29]. First, we applied the Maxfilter software (temporal extension of the signal space separation method, correlation window of 10 s, and correlation limit of 0.9) to remove external noise. Then we used FielTtrip software 28 to automatically scan the data for artifacts, which were visually confirmed by an MEG expert. Artifact-free data were segmented in 4-s epochs, plus 2 s of real data at each side as padding.

Afterwards, we estimated the source level activity for each individual. As source model, we used a 1 cm homogeneous grid of source positions defined in MNI space and labeled according to the automated anatomical labeling (AAL) atlas. This source model consisted of 1202 positions in 78 cortical areas and was transformed to subject space using a linear transformation between the template and the T1-weighted MRI of the participant. This image was also used to generate a single-shell head model defined by the inner skull surface. Then, we combined the head model, the source model, and the sensor definition to create a lead field using a modified spherical solution. As the last step, we used a linearly constrained minimum variance beamformer as inverse method.

We estimated FC by means of the phase locking value (PLV), a phase synchronization metric that evaluates the distribution of the phase difference between two-time series. Briefly, after source reconstruction, the dataset consisted of matrices of 1202 nodes by 4000 samples by epochs for each of the 4 frequency bands studied here. Then, for each frequency band and epoch, we calculated the PLV [30] via the following procedure: firstly, we used the Hilbert transform to extract the instantaneous phase *φ*_*j*_(*t*) for each node *j* = 1…1202 and time *t* = 1…4000 ms:

$${z}_{j}\left(t\right)={x}_{j}\left(t\right)+i\bullet Hilbert\left({x}_{j}\left(t\right)\right)={A}_{j}\left(t\right)\bullet {e}^{i{\varphi }_{j}\left(t\right)}$$

Secondly, we estimated the synchronization between each pair of signals $j$ and $k$ by means of their difference of phases ${\varphi }_{j}\left(t\right)$ and ${\varphi }_{k}\left(t\right)$ using the following expression:

$$PLV= \frac{1}{M}\left|\sum\limits_{m=1}^{M}{e}^{i\left({\varphi }_{j}\left({t}_{m}\right)-{\varphi }_{k}({t}_{m})\right)}\right|$$

where *T* = 4000 is the number of samples in the time series (4 s per epoch at 1000 Hz sampling rate). Lastly, we averaged the PLV matrices across epochs to obtain a more robust estimator of resting-state FC.

This algorithm provided symmetrical whole-brain matrices of 1202 × 1202 nodes per participant and frequency band (theta, between 4 and 8 Hz; alpha, between 8 and 12 Hz; beta, between 12 and 30 Hz; and gamma, between 30 and 45 Hz) [31]. Then, we calculated the nodal strength (also known as weighted global connectivity), which is defined for each node as the sum of its FC with the rest of the nodes. To account for the number of links, the strength of each node was then normalized by dividing the number of links connected to it. This procedure resulted in one brain map of normalized node strengths per each participant and frequency band.

### APOE genotype

Genomic DNA was extracted from 10 ml blood samples in ethylenediaminetetraacetic acid. Detection of *APOE* genotype was performed with TaqMan technology using an Applied Biosystems 7900 HT Fast Real-Time PCR machine (Applied Biosystems, Foster City, CA). See the genotyping method previously described in Cuesta et al. [32] for more information. All the sample was included independently of *APOE* genotype in the initial analysis. Then, to evaluate the potential moderation role of genotype, the participants were classified as *APOE* ɛ4 carriers and noncarriers (i.e., ɛ3ɛ3). Participants who presented less frequent allele combinations (i.e., ɛ2ɛ2, ɛ2ɛ3, ɛ2ɛ4, and ɛ4ɛ3ɛ4) were excluded from the sample.

### Statistical analyses

#### Functional connectivity strength (strength FC)

Cluster-based permutation test (CBPT) was carried out separately for each frequency band [33]. We defined a cluster as a set of spatially adjacent nodes that presented a significant partial correlation (Spearman correlation using age as a covariate, *p* < 0.001) in the same direction between the strength FC values and each DR variable. In this framework, a cluster can be considered as a functional unit. Only clusters including at least 1% of the grid (i.e., a minimum of 12 nodes) were considered. The Spearman rho values were transformed into Fisher *Z* values, and the cluster-mass statistics were computed as the sum of the *Z* values of all nodes within the cluster. The *p* value for each cluster was calculated in a nonparametric fashion, using a null distribution generated by the mass of the main cluster obtained over 5000 random permutations (shuffled versions) of the data [30]. Only those clusters that resulted significant (*p* < 0.05) after this step were considered in further analyses. Then, we used the average of the strength FC values of the members of the cluster to obtain a representative FC marker. Of note, this FC marker would be indicating that the global FC of the possible significant clusters appeared to be associated with memory performance.

### Seed-based analyses (seed link FC)

To examine whether the strength FC results were caused by global or region-specific effects, we performed complementary seed analyses, using the previous clusters as seeds. For it, we calculated the average FC of each source position with the sources in the cluster. Then, we repeated the statistical CBPT analysis using these seed-based FC values instead of the strength FC values.

### Correlations between FC and measures of white matter integrity and brain volume

We used the aforementioned cluster markers in subsequent correlation analyses with measures of AD-specific signatures. As to this, we used both the whole sample and a stratification of the cohort by diagnosis (MCI and CI). To account for multiple comparisons, the resulting *p* values were corrected using a false discovery rate (FDR). All statistical analyses were carried out using MATLAB R2020b (MathWorks Inc.).

### Moderation analysis

Additionally, we analyzed the impact of FC on DR focusing on the possible influence exerted by *APOE* genotype. Each group was divided into ɛ4 carriers and ɛ4 noncarriers to evaluate multiple regression analysis (using age and years of education as covariates), and we calculated the increase in variance explained after including the interaction into the model. Then, we examined the effect of FC on delayed recall scores in the *APOE* genotype subgroups and used the Johnson-Neyman technique to identify the threshold where the synchronization shows a dysfunctional pattern in CI (CI33/CI34) and MCI (MCI33/MCI34). Statistical analyses were carried out using Process Macro, an extension for SPSS that calculates X’s direct, indirect, and total effects on *Y* and unstandardized and standardized regression coefficients, standard errors, *t*, *p* values, and *R*^2^ for the models [34].

## Results

### Delayed recall is associated with decreased occipital beta frequency band FC in MCI and CI participants

**Fig. 1:**
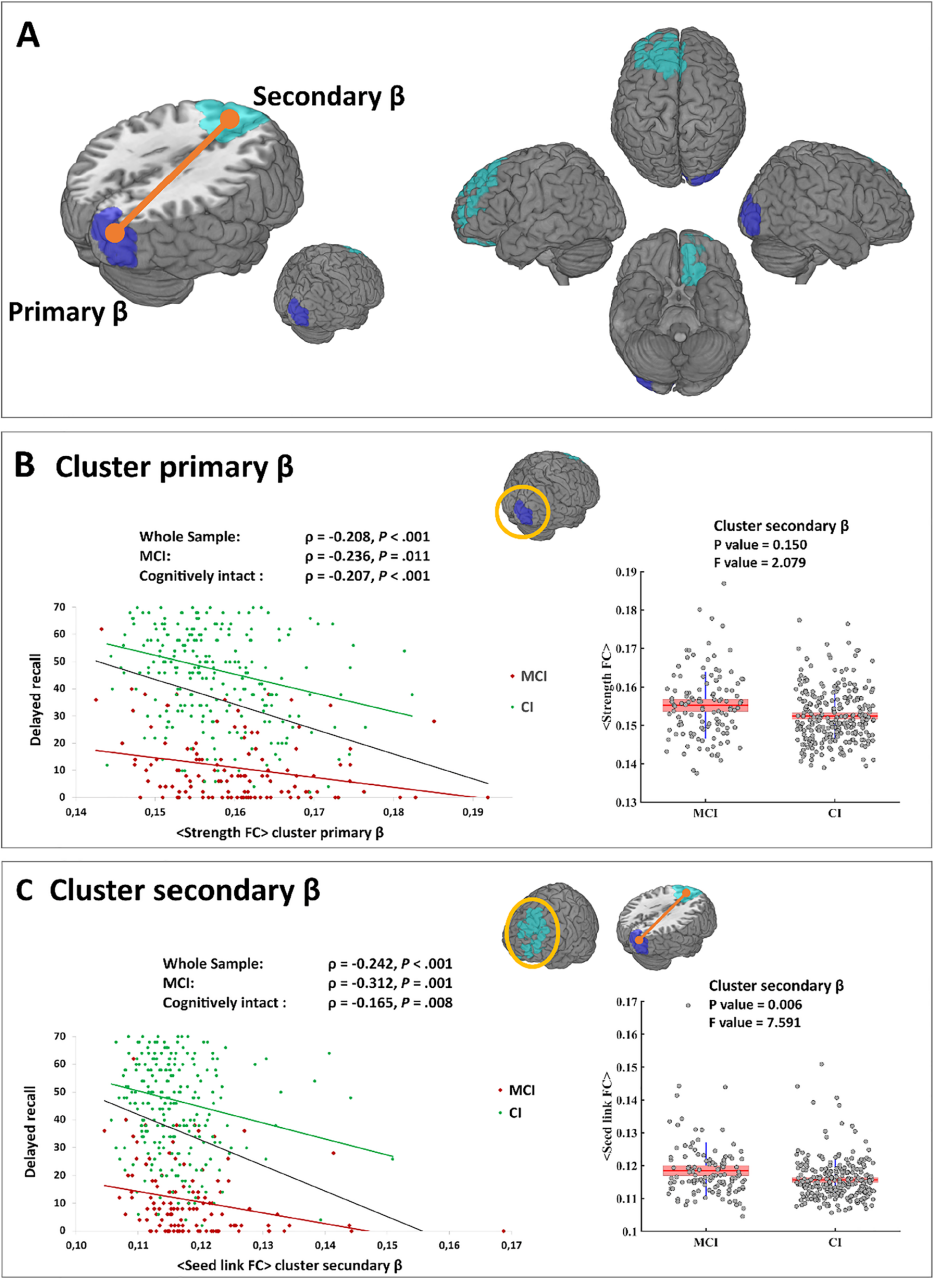
**A** In dark blue, marked as primary *β*, is displayed the brain region whose functional connectivity strength (strength FC) was found inversely correlated with delayed recall (DR). In light blue are depicted the region, marked as secondary *β*, whose FC with the primary *β* cluster was found to inversely correlate with DR. **B** Scatter plot shows the correlation between primary *β* cluster strength FC and DR computed with the whole sample (gray), MCI patients (red), and cognitively intact (CI) participants (green). Boxplot graphic shows the average strength FC of the primary *β* cluster for each group. **C** Scatter plot shows the correlation between primary *β* < – > secondary *β* FC and DR computed with the whole sample (gray), MCI patients (red), and cognitively intact (CI) participants (green). Boxplot graphic shows the average seed link FC of the secondary *β* cluster for each group. In boxplot graphics, vertical blue line indicates 95% confidence interval, whereas salmon boxes depict avg ± sd

One significant (CBPT *p* value = 0.019) primary cluster emerged in the beta band (primary *β*, Fig. 1A and B), located on the right occipital region (detailed regions within the cluster are shown in Table 2). The correlation between the strength FC of this cluster and DR performance was negative, indicating that the higher the strength FC, the lower the memory performance. In addition, the correlation remained significant when looking at the MCI (rho =  − 0.236; *p* = 0.011) and CI (rho =  − 0.207; *p* < 0.001) groups separately (Fig. 1B). The strength FC value was found to be higher in the MCI patients than in the CI individuals, but the difference did not reach significance (ANCOVA with age and education as covariates, *p* value = 0.150, *F* value = 2.079; see Fig. 1B).

**Table 2:** Main cluster and seed analyses presented increased in FC at less DR performance

Cluster	mβDR	%	sβDR	%
ROIs	Right middle occipital lobe	29.41	Left superior frontal gyrus. Medial	58.82
	Right cuneus	15.38	Left middle frontal gyrus	44.12
	Right superior occipital lobe	20.00	Left superior frontal gyrus	48.15
	Right inferior occipital lobe	40.00	Left cingulate gyrus. Anterior part	42.11
			Left superior frontal gyrus. Medial orbital	50.00
			Left gyrus rectus	37.50
			Left superior frontal gyrus. Orbital	66.67

Beta main cluster whose functional connectivity strength (FC) was significantly correlated with DR was used a seed in a seed-based analysis. List of regions of interests (ROIs) from the AAL atlas that were captured for each significant cluster. % depicts the percentage of the ROI that fall within the cluster (only show the ROIs above 15%)

When exploring the seed-based FC in the brain, using the primary *β* cluster as seed, we found one significant secondary cluster whose FC with the primary cluster showed to be negatively correlated with DR. The correlation between DR and the seed link FC remained significant when looking at the CI (rho =  − 0.165; *p* = 0.008) and MCI (rho =  − 0.312; *p* < 0.001) groups separately (Fig. 1A and C). However, the average seed link FC was significantly different between groups (ANCOVA with age and education as covariates, *p* value = 0.006, *F* value = 7.591), showing higher values for the MCI patients than for the CI individuals (Fig. 1C).

### Lower occipital beta FC is differently associated with brain structure in MCI and CI patients

To better understand our results, we explored the relationship between the strength FC and structural measures (see Table 3). About MCI, white matter (*r* =  − 0.24; *p* = 0.01) and gray matter volume (*r* =  − 0.22; *p* = 0.02) exhibited negative association with primary *β* electrophysiological activity. The same direction was observed with right cingulum in the hippocampal area (*r* =  − 0.25; *p* = 0.01) and forceps major fractional anisotropy (*r* =  − 0.24; *p* = 0.02). In contrast, only one inverse correlation was found for the FC of the link mβ-sβ related to the right gray matter volume (*r* =  − 0.19; *p* = 0.04). On the other hand, the CI group’s association in primary *β* with structural measures was only expressed inversely with forceps minor fractional anisotropy (*r* =  − 0.14; *p* = 0.03).

**Table 3:** The relationship between the strength FC and structural measures

	MCI			CI		
	Structure	*r*	*p* value	Structure	*r*	*p* value
mβDR	Total WM	− 0.2431	0.0086	Forceps minor	− 0.143	0.0253
	Right cingulum	− 0.2462	0.0122			
	Forceps mayor	− 0.2365	0.0161			
	Total GM	− 0.2152	0.0204			
sβDR	Right total GM	− 0.1923	0.0386			

Results for Spearman correlation analyses between the FC of the edge < main-β. seed2-β > and brain structural integrity scores. Total gray matter volume (GM) and total cerebral white matter volume (WM) in mm3; right cingulum along the hippocampal cortex fractional anisotropy; forceps major fractional anisotropy; forceps minor fractional anisotropy

### Moderation effect of APOE-ε4 genotype, beta FC link in delayed recall scores

**Fig. 2:**
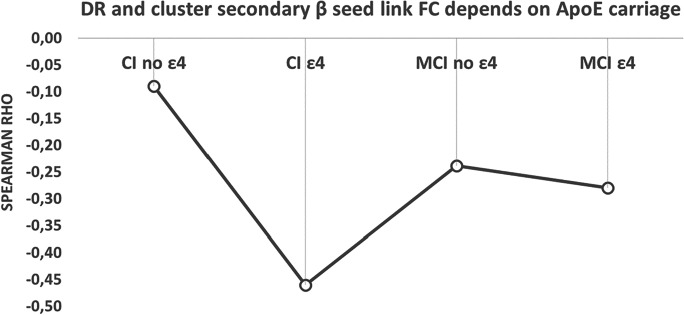
Variation of the Spearman rho scores (age and education as covariates) for the correlation between delayed recall (DR) and secondary *β* seed link FC with ε4 *APOE* carriage. In order to show qualitatively the dependence of *APOE* in the relationship between DR and the FC of the link primary *β* <—> secondary *β* clusters, we have depicted the Spearman rho scores (computed with age as covariate) obtained for four groups: CI/MCI with and without any allele *APOE *4. As it can be seen, the influence of the allele 4 of the *APOE* is notorious in the CI ε4 group. The solid black line that connects all groups is just indicative of the Rho’s variance

After we had described how lower levels of DR related to a distinctive strength FC profile, we analyzed the impact of *APOE* genotype as a moderator (*W*) between the pattern of FC in right occipital and left anterior areas in beta band (*X*) and the DR scores (*Y*) separately for each group. Our results showed a significant regression coefficient different from zero (b3 =  − 1428.07, *t* (209) =  − 3.10, *p* = 0.002) for XW only for the CI group, meaning that the effect of the network on episodic memory scores depends on the *APOE* genotype in cognitively intact participants. Once we defined the moderator effect of the *APOE *genotype, we tried to identify what was the threshold in FC that differentiated the episodic memory performance in carriers vs noncarriers. The Johnson-Neyman technique allowed the exact calculation of the conditions and the limit values in which a moderator obtains statistically significant slopes. Our results showed significant differences in FC values higher than 0.116, which means that the effect of *APOE *genotype on episodic memory scores was expressed only when the value of FC was above 0.116 (See Fig. 2).

## Discussion

Episodic memory dysfunction and altered functional connectivity patterns are among the most important features associated with cognitive decline in AD and its prodromal stages [5]. Together with the genetic risk posed by the presence of the ε4 allele of the *APOE* gene, these variables can provide valuable information on the identification of individuals with a higher risk of developing AD. With this aim, we evaluated these variables in a large sample of participants either healthy or diagnosed with MCI. Firstly, we analyzed the relationship of FC and delayed recall, considering, in a second step, the potential moderator effect of the *APOE* genotype on this relationship. Results from our analysis identified a cluster of right occipital regions whose global beta band functional connectivity were negatively associated with delayed recall scores. Moreover, a post hoc seed-based analysis showed that the left frontal cortex was the primary region contributing to this effect.

Previous studies have linked hypersynchronization in the beta band with early stages of pathological aging [10]. In task, there is evidence showing that MCI patients who later progress to AD present higher values of functional connectivity in the beta band than those who will remain stable [10]. Other studies have confirmed that these values can be a good predictor of progression from MCI to AD [35]. This hypersynchronization has been proposed to be derived from neuropathology. In particular, high accumulations of beta amyloid protein are especially toxic to inhibitory neurons, inducing an excess of excitability that can cause spurious synchronization (Garcia-Marin et al., [36]; see also Maestú et al., [17] for a multicentric study). In this line, our results reveal that an increase of functional connectivity between frontal and posterior regions is associated with reduced delayed recall scores. Of importance, this functional coupling was greater in the MCI group than in the healthy participants, according to the idea of a progressive increase of neuronal excitability along the continuum of the disease, until the network breakdown at AD [35]. Moreover, these results can also be interpreted as well as a hampered desynchronization. Beta desynchronization is one of the main electrophysiological features of resting state brain activity, and a higher FC is compatible with the idea of an interruption of the natural desynchronization process at rest, reflecting a dedifferentiation process [37]. Nevertheless, we will refer to our finding as hypersynchronization, as that is the direct interpretation of the actual results.

Interestingly, when we tested the potential role of the *APOE* ε4 allele as a moderator of the relationship between the identified pattern of FC (between right occipital and left anterior areas in beta band) and delayed recall memory, we found a significant effect in the healthy ε4 carriers. In this subsample, the indirect relationship between beta FC and memory performance was exacerbated, whereas in MCI patients, the role of *APOE* ε4 allele was nonsignificant, likely due to the more advanced stage of these patients in the AD continuum. These results would be indicating the existence, in the healthy ε4 carriers, of a selective vulnerability in the electrophysiological process of beta hypersynchronization, producing a result that mirrored the one observed in pathological aging. The deleterious effect of having one or two copies of the *APOE* ε4 allele has been largely described in the literature [38]. Hence, it does not come as a surprise that in ε4 carriers’ functional alterations in the brain are more strongly associated with cognitive outcomes than in noncarriers, most likely due to a reduced remodeling and repair capacity resulting from impaired lipid transportation processes in ε4 carriers. Unfortunately, genetic background is a non-modifiable risk factor for cognitive decline, and no therapy can reduce the associated risk directly. However, several modifiable lifestyle factors have been shown to exert an influence on FC [29, 39]. Given that this pattern of increased FC between right occipital and left anterior areas in beta band predicts lower cognitive performance in ε4 carriers, future studies looking into the potential effect of several lifestyle factors to decrease this pattern of FC are warranted.

Episodic memory performance has been traditionally understood as the first clinical manifestation of an underlying Alzheimer-type neuropathological process [3–5]. However, the emergence of clinically measurable memory damage occurs in a stage where the brain damage is substantial. Here, we sought for functional integrity markers associated with memory performance that can be used as proxies of the underlying neuropathological process. Since the FC markers can be computed in every participant, this approximation would allow the identification of individuals with a higher risk of developing AD [40, 41]. Here we found that beta frontal-occipital hypersynchronization predicts a poor DR performance in healthy and pathological aging. In addition, this FC marker seemed to be more pronounced in MCI patients. A similar finding was reported by Canuet et al. [42] in an independent sample with abnormal CSF p-tau levels. Furthermore, in a longitudinal study, beta FC was negatively associated with working memory and executive function, and, at the baseline, their levels were the highest in progressive MCI compared to stable MCI, showing a high accuracy (71%) to discriminate between the two groups [35]. A work similar to ours showed that an increase in beta and gamma frequencies above 16 Hz correlated with lower cognitive performance [43]. Finally, the augmentation of beta-band FC with age in healthy aging has been described in a large study [44]. In addition to these FC patterns, many works have shown the inverse relationship of beta power with working memory over parietal sites [45, 46] and with immediate and delayed recall in posterior areas [47], supporting the idea of a hampered beta desynchronization at rest is associated with these domains.

When evaluating the relationship between the FC markers and brain structural integrity, the MCI patients showed a negative association between occipital FC with white and gray matter measures, reinforcing the relationship between the FC markers described in this study with the progression of the dementia. This points out that the worsening of the neuropathology in these patients would be captured by their even higher hypersynchronization when compared to controls. This endorses our view of the FC values here reported as possible markers that would help tracking the trajectory a specific patient along the AD continuum.

Considering all the evidence shown so far, a tentative explanation of the present findings is that the hypersynchronization in beta FC reflects a progressive deterioration of neuronal function that advances at hand with the evolution of the neuropathological process associated with dementia. In healthy aging, the key factor was the presence of the ε4 allele; while the inverse relation between FC and DR was clearly significant in healthy ε4 allele carriers, it did not reach significance in noncarriers. On the other hand, the MCI patients showed an exacerbation of this malfunction both in ε4 carriers and noncarriers. This process could be understood as maladaptive process of dedifferentiation [37]. The advance of the neuropathology would be altering the ability of the brain to focus its activity, reflected as a lack of beta desynchronization. This would be likely to the lack of inhibitory connection shown in the pathological process related to AD [42, 48, 49]. Moreover, higher synchronization was not associated to better cognitive performance, discarding the classical interpretation as a compensatory mechanism. It is interesting to note here that the occipital lobe has been usually less impaired until the last state of the AD, and the appearance of the beta cluster in our results could be explained by the effect of amyloid pathology in the hippocampus regions that results in a reduction in neuronal input to occipital areas. This decrease that influences the brain organization at the functional level is defined as a functional diaschisis [50].

Finally, some limitations of our study should be addressed in future research. The use of a cross-sectional design provides only a snapshot of brain activity, and the picture can be enhanced by the developing of longitudinal studies aimed to track the individual FC changes along the AD continuum. In addition, the combination of these techniques with neuropathological markers of AD, as amyloid or tau pathology, would improve the clarity of the results since we would be able to distinguish whether these results are specific of AD [51].

Nevertheless, our results strongly suggest that the lack of desynchronization (or hypersynchronization) in beta band has the potential to be used for assessment of pharmacological and non-pharmacological interventions. Moreover, the results can be transferred from the costly MEG to the widely available EEG. Altogether, they have the potential to become a widely used noninvasive biomarker of the neuropathological progression underlying the developing of dementia.

## Conclusion

The goal of the present study was to bridge the gap between measures of cognitive performance and electrophysiological markers in healthy and pathological aging. The results suggest that beta hypersynchronization-associated markers could be useful to evaluate brain health. This contrasts with the typical use of RS markers more focused in the assessment of alpha band features. Here, our results indicate that the brain seems to progressively loss the ability to desynchronize beta FC and that this process would be starting to become significant in preclinical stages, at least in participants with AD risk factors such as the presence of the *APOE* ε4 allele.
